# Validation of the SQUASH Physical Activity Questionnaire in a Multi-Ethnic Population: The HELIUS Study

**DOI:** 10.1371/journal.pone.0161066

**Published:** 2016-08-30

**Authors:** M. Nicolaou, M. G. J. Gademan, M. B. Snijder, R. H. H. Engelbert, H. Dijkshoorn, C. B. Terwee, K. Stronks

**Affiliations:** 1 Department of Public Health, Academic Medical Centre, University of Amsterdam, Amsterdam, The Netherlands; 2 Education of Physical Therapy, Amsterdam School of Health Professions (ASHP), University of Applied Sciences, Amsterdam, The Netherlands; 3 Department of Rehabilitation, Academic Medical Center (AMC), Amsterdam, The Netherlands; 4 Department of Epidemiology and Health Promotion, Public Health Service of Amsterdam, Amsterdam, The Netherlands; 5 Department of Epidemiology and Biostatistics and the EMGO Institute for Health and Care Research, VU University Medical Center, Amsterdam, The Netherlands; Vanderbilt University, UNITED STATES

## Abstract

**Purpose:**

To investigate the reliability and validity of the SQUASH physical activity (PA) questionnaire in a multi-ethnic population living in the Netherlands.

**Methods:**

We included participants from the HELIUS study, a population-based cohort study. In this study we included Dutch (n = 114), Turkish (n = 88), Moroccan (n = 74), South-Asian Surinamese (n = 98) and African Surinamese (n = 91) adults, aged 18–70 years. The SQUASH was self-administered twice to assess test-re-test reliability (mean interval 6–7 weeks) and participants wore an accelerometer and heart rate monitor (Actiheart) to enable assessment of construct validity.

**Results:**

We observed low test-re-test reliability; Intra class correlation coefficients ranged from low (0.05 for moderate/high intensity PA in African Surinamese women) to acceptable (0.78 for light intensity PA in Moroccan women). The discrepancy between self-reported and measured PA differed on the basis of the intensity of activity: self-reported light intensity PA was lower than measured but self-reported moderate/high intensity PA was higher than measured, with wide limits of agreement. The discrepancy between questionnaire and Actiheart measures of moderate intensity PA did not differ between ethnic minority and Dutch participants with correction for relevant confounders. Additionally, the SQUASH overestimated the number of participants meeting the Dutch PA norm; Cohen’s kappas for the agreement were poor, the highest being 0.30 in Dutch women.

**Conclusion:**

We found considerable variation in the test-re-test reliability and validity of self-reported PA with no consistency based on ethnic origin. Our findings imply that the SQUASH does not provide a valid basis for comparison of PA between ethnic groups.

## Introduction

Physical activity is an important determinant of health [1]. In the Netherlands recent figures indicate that 61% of the population meets the Dutch healthy Physical Activity (PA) guideline of 30 minutes moderate intensity PA daily [2]. However, in ethnic minority and migrant groups this percentage is much lower, 45%, 49% and 56% in Turkish, Moroccan and Surinamese respectively [3]. Hence, low PA may contribute to the disparities in health observed among these populations.

Physical activity levels are often evaluated on the basis of self-report [4]. Questionnaires are the preferred method for assessing in large-scale monitoring and observational studies due to their low cost compared to accelerometers or other objective measures and because they provide detailed estimates of the contexts and domains where PA takes place. However, they are prone to measurement error and bias due to misreporting, either deliberately (due to social desirability) or because of difficulties with remembering and estimating the frequency and duration of different activities [5, 6]. In a multi-ethnic population additional bias may arise if particular constructs are interpreted differently by different groups, or if certain groups are more likely to provide socially desirable answers [6–8]. Bias may also arise due to the use of items that are more suited to one group’s language and/or cultural experiences, for example questions about cycling. Additionally, participants with low PA levels (as is presumably the case among ethnic minorities) may have less structured PA patterns and thus have more difficulty with recalling activities [9].

The SQUASH (Short QUestionnaire to ASsess Health enhancing physical activity) is a commonly used instrument in the Netherlands to assess PA, particularly in surveillance studies [3]. It was developed by the Dutch National Institute of Public Health and the Environment (RIVM) to measure PA with respect to occupation, leisure time, household, transportation means, and other daily activities. The SQUASH was designed to give an indication of the habitual activity level and was structured in such a way that it would be possible to assess compliance to physical activity guidelines. [10] While it has been shown to be valid in measuring PA among the Dutch population [10, 11], its reliability and validity among ethnic minority groups has not been established.

We aimed to evaluate the test-re-test reliability and construct validity of the SQUASH questionnaire in measuring self-reported habitual PA in a multi-ethnic population using a combined accelerometer and heart rate measurement device (Actiheart). We expected that the SQUASH would be relatively reliable (i.e. would perform consistently) in all groups. However, as the SQUASH was designed for the Dutch population, we expected that construct validity may be lower in the ethnic minorities.

## Methods

### Study population

We included participants of the baseline examination of the HELIUS study (Healthy Life in an Urban Setting), a multi-ethnic cohort study that included adults aged 18–70 years in Amsterdam, the Netherlands [12]. The HELIUS study protocols were approved by the AMC Ethical Review Board, and all participants provided written informed consent. The SQUASH validation study aimed to include 100 (50 men and 50 women) persons per ethnic group: African Surinamese, South Asian Surinamese, Moroccan, Turkish and Dutch origin. This was based on the COSMIN (COnsensus-based Standards for the selection of health Measurement INstruments) criteria which indicates that for a validation study a sample size of 30–49 participants is considered “fair,” while 50–99 participants is considered “good” [13]. Of 1077 persons invited to participate 151 were not able to be contacted for an appointment, 92 were not eligible for the study and 356 persons refused to participate. Of the participants, 4 did not wear the Actiheart for the full five days, 8 had either ‘bad’ or incomplete data and 4 had an ‘other’ ethnic origin. Thus 462 participants were included in the analysis of validity. Of these, 119 persons did not complete the SQUASH a second time, resulting in 343 participants in the reliability study.

### Study procedure

The study took place from November 2012 to November 2013. HELIUS participants who indicated willingness to participate in sub-studies as part of the informed consent procedure were sent an invitation letter and brochure explaining the aims of the study and study procedures, followed by a telephone call within 2 weeks. During this call, individuals were screened for eligibility. Exclusion criteria were, 1) inability to complete the calibration of the Actiheart, i.e. chest pain upon exertion or difficulties in climbing stairs due to cardiac reasons; 2) use of medication that might interfere with heart rate measurements (β-blockers); 3) pregnancy.

Participants were invited to the study location for the placement of the Actiheart and were sent the SQUASH questionnaire for completion prior to their appointment. On the day of their appointment, participants were requested to avoid ingesting a heavy meal and to abstain from coffee, alcohol and cigarettes for 3 hours, as these substances may influence heart rate measurements and thus, the calibration study. During the visit to the study location we calibrated the Actiheart (see below for description of this procedure) and set it up for long-term recording. Participants were asked to wear the Actiheart continuously for a five day period during free-living (including at least one weekend day), after which they returned it to the research location.

Participants also received a *second* copy of the SQUASH with instructions to complete it four weeks after their appointment. Postage-paid envelopes were provided for the return of the questionnaire. We provided travel compensation in the form of a gift card and, upon receipt of the second copy of the SQUASH, participants received personalised feedback based on the Actiheart measurements.

### Self-reported physical activity (SQUASH)

Physical activity was measured using the SQUASH questionnaire [14]. The SQUASH includes questions on multiple activities referring to a ‘normal’ week in recent months: actively commuting (walking, cycling), physical activity at work or school, household activities, leisure time activities (sports, walking, gardening, cycling). Respondents were asked to fill in how many days a week they engaged in the activities, the average time per day spent on each activity (hours and minutes) and the intensity at which they did the activity (low, moderate, high). Missing and unlikely values were handled according to standardised methodology described in the user manual that accompanies the SQUASH, as described by Wendel-Vos et al.[14]. Briefly, results from the SQUASH were converted to minutes per week spent in light and moderate/high intensity activities based on age-specific Metabolic Equivalent Tasks (METs) derived from Ainsworth’s compendium of physical activity [15].The Dutch PA norm (NNGB) includes different cut-points for moderate- and high intensity activities for adults younger than 55 years (4.0 and 6.5 METs, respectively) and adults aged 55 years and older (3.0 and 5.0 METs, respectively) [2]. We categorized participants according to whether they met the NNGB by summing up the number of days per week for each moderate and high intensity activity lasting at least 30 minutes. A minimum of 5 days resulted in participants being categorized as achieving the NNGB.

The questionnaire was available in Dutch and was also translated to Turkish (using forward and back-translation techniques). Surinamese people generally speak the Dutch language fluently while the main language used among Moroccan people in the Netherlands is Berber, which is not a written language, thus the SQUASH was not translated for these two groups. Based on previous experience we expected that at least 70% of Moroccan participants could complete the SQUASH in Dutch. We aimed to test the validity of the questionnaire as it is intended for use, i.e. self-completed, thus we did not offer help with filling it in, however, it is possible that some participants received help from family members.

### Objectively measured physical activity

Objectively measured physical activity was assessed using Actiheart devices (version 4, CamNtech Ltd, UK). The Actiheart is a chest-worn monitoring device that records heart rate and physical activity in order to measure activity energy expenditure. Brage et al [16] reported that Actiheart measures of movement and heart rate generally agreed well with criterion measures of acceleration and heart rate: the linear relationship between movement and acceleration was strong (R^2^ = 0.99, P<0.001) and correlations with activity intensity were high (R^2^>0.84, P<0.001). During the visit to the study location the Actiheart was individually calibrated for each participant using a *sub-maximal* linear step test. Details of this step test have previously been described [16] but briefly: the step test consists of 8 min of stepping up and down a 200mm high step, starting with one foot placement per second for the first minute, steadily increasing and followed by a 2 minute recovery phase. Upon completion of the step test ECG signals from the Actiheart were downloaded into the manufacturer’s software. The Actiheart was placed in the position with the most optimal ECG signal (established prior to conducting the step test) and participants were instructed on to wear the device for five full days, including the day of placement.

Upon return of the Actiheart the long-term data were downloaded and processed in the manufacturer’s software. This involved an initial cleaning step, followed by a branched equation algorithm to translate the accelerometer and heart rate data into an estimate of minutes per day spent in several MET-categories [17]. We included data from 4 x 24 hour days per participant; the day that the Actiheart was placed was not included as it did not cover a full 24 hour period. Incomplete days and data of participants that did not wear the Actiheart for 4 days were excluded from further analysis (see method section for specification of numbers excluded).

For each complete day measured, the minutes of moderate- and PA were determined by using age adjusted MET values as described in the questionnaire section above. Light Intensity PA was calculated as all activity between 1.5 < 4.0 METs for the younger age group, and 1.5 < 3.0 METs for the older age group. In order to compare Actiheart outcomes (minutes per day) with the SQUASH, which derives minutes per week we calculated the average minutes per day of the days recorded and multiplied this by 7. The NNGB was calculated as follows: If the amount of time spent in moderate and high intensity PA was at least 30 minutes on a single day then those days were summed for determining the NNGB. Participants were categorized as achieving the NNGB when the fraction of a week was 5/7 of the total number of days measured (i.e. 3 of the 4 days in this study as we only included 4 full days of Actiheart measurements).

### Other variables measured

Potential covariates (Ethnicity, migration generation level, BMI, waist circumference and educational level) included in the analyses were obtained from the HELIUS study baseline data, as described by Stronks et al [12].

A participant was considered as being of non-Dutch ethnic origin if at least one of his parents was born abroad. Additionally, Surinamese subgroups (South-Asian or African) were classified according to self-reported ethnic origin. Generation status was defined on the basis of birthplace. Those born outside the Netherlands and having at least one parent born outside the Netherlands were considered first generation migrants, those born in the Netherlands and having one or both parents born abroad were considered second generation [18]. Trained research assistants measured weight and height in duplicate in barefoot subjects wearing light clothes only. Waist circumference was measured using a tape measure at the level midway between the lowest rib margin and the iliac crest. Body mass index (BMI) was calculated as weight (kg) divided by height squared (m2) [19]. Participants’ highest completed education was categorized as “never been to school or elementary schooling only”, “lower vocational schooling or lower secondary schooling”, “intermediate vocational schooling or intermediate/higher secondary schooling (general)”, and “higher vocational schooling or university”, at the time of filling in the HELIUS questionnaire.

Social desirability was examined using the Social Desirable Response Set (SDRS) from Hays et al [20]. The SDRS consists of a set of five propositions, scored using a 5-point Likert scale (e.g. I am always courteous even to people who are disagreeable). Participants receive 1 point per question for the most extreme score–with question 1 and 5 scoring ‘opposite’ to the other 3 questions. Thus scores range from 0 to 5 points–with 5 representing a high tendency to provide socially desirable answers.

### Analysis

#### Test-re-test reliability

Participants filled in the SQUASH twice, with an interim period of 4 weeks. Only participants who filled in the second questionnaire within the required time (maximum 12 weeks after the first) were included. We expected that this period was long enough to ensure that participants could not recall what they filled in the first time and short enough to prevent large changes in physical activity levels. For continuous measures (min/week engaged in light and moderate/high intensity physical activity) we calculated Intraclass Correlation Coefficients (ICC) using a two-way random effects model for absolute agreement. ICCs higher than 0.7 were considered strong. We expected reliability coefficients to be in the range of 0.6–0.7, as was found in a recent review of reliability of PA questionnaires [5]. Additionally we calculated the 95% Limits of Agreement between subjective and objective PA as a parameter of measurement error [21].

#### Construct validity

Spearman’s correlations were calculated to compare the minutes per week spent in light and moderate/high intensity physical activity as measured by the Actiheart and the SQUASH (filled in at the first time point). Validity coefficients were expected to be higher than 0.5 [22]. As the SQUASH is designed with the Dutch population in mind, we expected that the validity coefficients would be lower among the ethnic minority groups. We used the Bland–Altman method to evaluate bias, 95% limits of agreement were used for describing the total error between the two methods. The Actiheart values were regarded as the ‘standard’ against which the difference between the two measures was compared [21]. Differences at the individual level between the two measures of moderate to high intensity PA should in theory be within range of the NNGB which specifies that persons are sufficiently active if they engage in moderate to high intensity PA for at least 30 minutes per day, five days per week.

For the dichotomous measure, ‘fulfils NNGB’, in both the reliability and construct validity analysis we assessed Cohen’s Kappa where kappa < 0.20 was considered ‘poor’, 0 to 0.20 ‘slight’, 0.21 to 0.4 ‘fair’, 0.41 to 0.60 ‘moderate’, 0.61 to 0.8 ‘substantial’, and above 0.81 ‘almost perfect’ [23].

#### Ethnic differences

Ethnic differences between objective (Actiheart) and subjective (SQUASH) measures were studied using linear regression with the Dutch group as reference accounting for determinants of PA (education level and BMI) as well as for relevant confounders (age and social desirability).

Analysis was conducted with IBM SPSS 20.

## Results

The characteristics of participants are shown in Table 1. The average age (SD) of participants ranged from 40.2 (9.6) years among Turkish women to 53.0 (6.5) years among Dutch origin men. Among the ethnic minority groups, the majority were first generation migrants (78 to 97%). Body weight varied amongst the different sub-groups, the highest mean BMI amongst men was observed in Turkish participants, while amongst women this was observed in African Surinamese (28.5 (3.9) and 30.7 (7.0) respectively). Educational level also differed per ethnic group, with Dutch origin participants having completed a higher level of education than the ethnic minority groups; 62–66% had a high education level compared to 15% among Turkish men and 17% among Moroccan women. The ethnic minority groups scored higher on social desirability (range 1.3–2.0 out of 5 points) compared to Dutch origin participants who scored between 0.7–1.0.

**Table 1:** Participant Characteristics.

	Dutch		South Asian Surinamese		African Surinamese		Turkish		Moroccan	
	Men n = 44	Women n = 67	Men n = 43	Women n = 55	Men n = 49	Women n = 42	Men n = 48	Women n = 40	Men n = 37	Women n = 37
**Age in years, mean (SD)**	53.0 (6.5)	47.2 (9.6)	48.9 (9.3)	45.5 (10.0)	51.5 (6.8)	51.4 (5.7)	43.7 (9.8)	40.2 (9.6)	40.6 (11.8)	42.4 (9.9)
**1st generation, n (%)**	-	-	38 (90.5)	48 (87.3)	45 (97.5)	39 (95.1)	39 (78.0)	37 (86.0)	29 (78.4)	30 (81.1)
**BMI (kg/m**^**2**^**), mean (SD)**	25.4 (3.7)	24.0 (3.7)	26.3 (4.1)	26.1 (4.7)	25.6 (3.8)	30.7 (7.0)	28.5 (3.9)	28.7 (5.8)	26.6 (3.6)	28.0 (5.6)
**Waist circumference, cm (SD)**	96.7 (11.6)	85.5 (10.9)	95.6 (10.7)	87.4 (10.5)	92.1 (10.4)	98.1 (14.6)	98.1 (10.5)	90.7 (13.1)	94.9 (9.3)	90.7 (12.1)
**Highest education, n (%)**										
• **1 (low)**	0	1 (1.5)	7 16.7)	8 (14.8)	2 (4.4)	4 (9.8)	10 (20.8)	11 (26.2)	5 (14.3)	14 (38.9)
• **2**	6 (13.3)	9 (13.4)	17 (40.5)	20 (36.4)	22 (47.8)	14 (34.1)	21 (43.8)	5 (11.9)	10 (28.6)	3 (13.9)
• **3**	11 (24.4)	13 (19.4)	8 (19.0)	16 (29.1)	10 (21.7)	13 (31.7)	10 (20.8)	18 (42.9)	14 (40.0)	11 (30.6)
• **4 (high)**	28 (62.2)	44 (65.7)	10 (23.8)	11 (20.0)	12 (26.1)	10 (24.4)	7 (14.6)	8 (19.0)	6 (17.1)	6 (16.7)
**Social desirability score, mean (SD)**	0.7 (0.9)	1.0 (1.1)	1.6 (1.6)	1.7 (1.4)	1.8 (1.6)	2.0 (1.6)	1.4 (1.5)	1.3 (1.4)	1.5 (1.4	1.7 (1.1)
**Net response rate (%)**	61	64	41	60	54	51	50	26	31	33
**Completed SQUASH at two time points (%)**	89	84	74	74	72	72	70	63	57	57
**Test-re-test timespanMean weeks (SD)**	6.1 (3.3)	7.1 (4.2)	5.8 (2.3)	6.4 (2.6)	6.9 (4.2)	6.6 (4.4)	7.1 (3.8)	6.3 (3.2)	6.0 (3.8)	6.9 (2.3)

Highest education level: 1 = “never been to school or elementary schooling only”, 2 = “lower vocational schooling or lower secondary schooling”, 3 = “intermediate vocational schooling or intermediate/higher secondary schooling (general)”, and 4 = “higher vocational schooling or university”

The response rate differed between ethnic groups, and was lowest in the Turkish women (26%) and Moroccan men (31%). Information from the HELIUS study enabled us to compare responders and non-responders on main characteristics (full data not shown). Statistically significant differences between responders and non-responders, per ethnic group were as follows: Among Dutch, participants had a lower BMI than non-responders. Among South Asian Surinamese and Turkish, more women participated than men. Among African-origin Surinamese and Moroccan more first generation persons participated. No other differences between groups were observed.

Table 2 presents the results of the test-re-test reliability of the SQUASH. The average test-re-test time span was 6.5 weeks. Reliability varied greatly with no consistent patterns on the basis of ethnicity or the outcome variable used. The ICCs for continuous measures ranged from extremely low (0.05 for moderate/high intensity PA in African Surinamese women) to acceptable (0.78 for light intensity PA in Moroccan women, 0.64 for moderate/high intensity PA in Dutch women). Participants reported higher PA at the second time point for all continuous variables, as is reflected in the negative mean differences in the tables; Moroccan men were the only exception, reporting lower light intensity PA at the second time point. The limits of agreement between the measures at the two time points were wide for all groups. For meeting the norm for PA (NNGB) Cohen’s kappa ranged from poor (0.01 among South Asian Surinamese men) to, at best moderate (0.38 in African Surinamese men).

**Table 2:** Test-re-test reliability of the SQUASH questionnaire.

		Light intensity PA		Moderate and High intensity PA		Meets Norm PA
		Intra Class Correlation (95% CI)	Mean difference (min/week) ±95% Limits of Agreement	Intra Class Correlation (95% CI)	Mean difference (min/week) ±95% Limits of Agreement	Cohen’s kappa (95% CI)
**Women**	**Dutch n = 57**	0.41 (0.17, 0.60)	-360 ±2530	0.64 (0.39, 0.79)	-184 ± 906	0.29 (0.04, 0.55)
	**SA Sur. n = 42**	0.20 (0.00, 0.48)	-549 ±2583	0.32 (-0.27, 0.63)	-693 ± 2656	0.38 (0.13, 0.63)
	**African Sur. n = 31**	0.46 (0.12, 0.70)	-400 ±3510	0.05 (0.54, 1.06)	-843 ± 4721	0.18 (-0.16, 0.52)
	**Turkish n = 27**	0.21 (0.00, 0.54)	-912 ±3290	0.45 (-0.20, 0.75)	-210 ± 1305	0.35 (0.03, 0.68)
	**Moroccan n = 21**	0.78 (0.52, 0.91)	-475 ±1372	0.07 (-1.35, 0.63)	-770 ± 2561	0.07 (-0.20, 0.34)
**Men**	**Dutch n = 41**	0.56 (0.31, 0.74)	-115 ±1630	0.38 (-0.16, 0.67)	-578 ± 2198	0.04 (-0.22, 0.30)
	**SA Sur. n = 32**	0.51 0.19, 0.73)	-235 ±2240	0.37 (-0.31, 0.70)	-558 ± 2596	0.01–0.32, 0.32)
	**African Sur. n = 36**	0.46 (0.15, 0.68)	-694 ±2337	0.61 (0.23, 0.80)	-662 ± 1800	0.48 (0.23, 0.73)
	**Turkish n = 35**	0.40 (0.76, 0.64)	-1019 ±3357	0.35 (-0.29, 0.67)	-526 ± 2578	0.32 (0.03, 0.62)
	**Moroccan n = 21**	0.21 (0.00, 0.58)	390 ±2637	0.58 (-0.04, 0.83)	-307 ± 4653	0.12 (-0.25, 0.50)

SA Sur. = South Asian Surinamese, African Sur. = African Surinamese

Construct validity for light and moderate/high intensity PA is presented in Table 3. Objectively measured light intensity was on average higher than what was reported using the SQUASH in all groups. At the population level, mean differences between the two measures of light intensity PA ranged from 1053 minutes/week (150 min/day) in Dutch men to 3144 minutes/week (449 min/day) in Turkish women. The 95% limits of Agreement were wide (ranging from ±2930 to ±4215 min/week). Bland-Altman plots indicate that differences between the measures were larger with higher duration of objectively measured activity (see Figs A and B in S1 File “Bland Altman Plots for Light Intensity Physical Activity” in men and women respectively.

**Table 3:** Construct validity–differences between objective (Actiheart) and subjective (SQUASH) levels of Physical Activity.

		Actiheart, mean min/week (SD)	SQUASH, mean min/week (SD)	Mean difference (Actiheart–SQUASH), mean min/week (SD)	95% Limits of Agreement (min/week)	Spearman’s correlation coefficient (p-value)
**Light Intensity PA**						
**Women**	**Dutch (n = 67)**	3712 (1328)	1926 (856)	1788 (1675)	± 3283	-0.11 (0.374)
	**SA Sur. (n = 55)**	4021 (1594)	1510 (892)	2530 (2061)	± 4039	-0.28 (0.039)
	**African Sur. (n = 42)**	3259 (1379)	1799 (1459)	1468 (2169)	± 4215	-0.12 (0.437)
	**Turkish (n = 40)**	4612 (1418)	1436 (797)	3144 (1519)	± 2977	0.14 (0.407)
	**Moroccan (n = 37)**	4081 (1041)	1275 (1086)	2738 (1564)	± 3065	-0.13 (0.459)
**Men**	**Dutch (n = 44)**	3003 (1373)	1936 (966)	1053 (1495)	± 2930	0.20 (0.184)
	**SA Sur. (n = 43)**	3626 (1647)	1487 (976)	2165 (1853)	± 3631	0.05 (0.743)
	**African Sur. (n = 49)**	3795 (1754)	1040 (889)	2733 (1886)	± 3696	0.15 (0.312)
	**Turkish (n = 48)**	4237 (1683)	1196 (1143)	3049 (1756)	± 3088	0.40 (0.005)
	**Moroccan (n = 37)**	4490 (1730)	1310 (1036)	3181 (2115)	± 4145	-0.09 (0.609)
**Moderate and High Intensity PA**						
**Women**	**Dutch**	344 (274)	394 (396)	-52 (417)	± 818	0.41 (0.001)
	**SA Sur.**	288 (497)	354 (485)	-70 (702)	± 1376	0.15 (0.278)
	**African Sur.**	462 (1077)	443 (622)	8 (1259)	± 2467	0.34 (0.029)
	**Turkish**	222 (157)	373 (568)	-162 (604)	± 1184	0.30 (0.039)
	**Moroccan**	381 (539)	136 (206)	251 (522)	± 1023	0.12 (0.495)
**Men**	**Dutch**	489 (418)	413 (463)	68 (633)	± 1240	0.17 (0.277)
	**SA Sur.**	256 (306)	520 (657)	-265 (736)	± 1443	0.26 (0.091)
	**African Sur.**	286 (268)	414 (576)	-124 (598)	± 1172	0.38 (0.007)
	**Turkish**	271 (337)	467 (828)	-195 (804)	± 1575	0.08 (0.605)
	**Moroccan**	312 (320	476 (666)	-165 (682)	± 1337	0.18 (0.301)

SA Sur. = South Asian Surinamese, African Sur. = African Surinamese

Objectively measured moderate/high intensity PA was lower than was reported using the SQUASH. At the population level, mean differences between the two measures were, amongst Moroccan women (mean difference 251 minutes/week, 36 min/day) and Dutch men (mean difference 68 minutes/week, 10 min/day). African Surinamese women were the only exception (mean difference 8 minutes/week, 1 min/day). Although the differences between the Actiheart and SQUASH measures of moderate/high intensity PA were smaller than they were for low intensity PA, wide limits of agreement in all groups, indicate poor agreement between SQUASH and Actiheart measures at the individual level.

Finally, Spearman’s correlations between the Actiheart and SQUASH measures of PA varied per group and were smaller than expected; ranging from 0.08 for moderate/high intensity PA in Turkish men, to 0.41 for moderate/high intensity PA in Dutch women.

Table 4 shows the results of the analysis of the ethnic differences in the discrepancy between the objective and subjective measures of light intensity and moderate/high intensity PA. Among women, differences with the Dutch reference group in light intensity PA seem to be explained by differences in educational level, except in Turkish women, where the differences persisted even after additional adjustment for BMI and social desirability (Beta 1135 (95%CI 325, 1945)). Differences in moderate and high intensity PA were only statistically significant in Moroccan women. Among men, significant differences with Dutch men were observed in light intensity PA and these persisted after full adjustment, with the exception of South Asian Surinamese men (Beta 464 (95%CI -311, 1239)).

**Table 4:** Ethnic differences in the discrepancy between objective (Actiheart) and subjective (SQUASH questionnaire) measures of Physical Activity in minutes per week.

		Difference in Light Intensity PA			Difference in Moderate and High Intensity PA		
		Age adjusted	Age + Education	Fully adjusted	Age adjusted	Age + Education	Fully adjusted
**Women**	**Dutch origin**	Ref.	Ref.	Ref,	Ref.	Ref.	Ref.
	**SA Sur.**	905 (250, 1559)**-	589 (-113, 1291)	715 (-4, 1434)	-33 (-297, 231)	86 (-200, 372)	117 (-176, 409)
	**African Sur.**	135 (-850, 580)	-309 (-1043, 423)	9 (-800, 782)	34 (-254, 323)	111 (-187, 409)	130 (-192, 453)
	**Turkish**	1312 (580,2044)**	938 (156,1720)*	1135 (325,1945)*	99 (-394, 196)	-15 (-333, 303)	-43 (-373, 287)
	**Moroccan**	958 (215, 1700)*	463 (-349,1277)	653 (-185,1490)	307 (7, 606)*	407 (76, 738)*	417 (76, 758)*
**Men**	**Dutch origin**	Ref.	Ref.	Ref,	Ref.	Ref.	Ref.
	**SA Sur.**	728 (13, 1443)*	500 (-113, 1291)	464 (-311, 1239)	-310 (-599, -21)*	-224 (-527, 79)	-239 (-549, 71)
	**African Sur.**	1504 (818,2191)**	1303 (584, 2021)**	1286 (552, 2019)*	-198 (-476, 80)	-88 (-374, 198)	-106 (-399, 187)
	**Turkish**	1290 (572,2007)**	1029 (251,807)*	1126 (313,1939)*	-195 (-485, 95)	-108 (-418, 203)	-109 (-434, 216)
	**Moroccan**	1236 (445,2027)**	1058 (230,1886)*	1083 (239,1927)*	-143 (-463, 177)	-83 (-413, 247)	-92 (-429, 245)

Fully adjusted model included age, BMI, highest education level and Social desirability score. Statistically significant differences between ethnic groups and Dutch men/women (*p<0.05). (**p<0.01)

Unstandardized betas shown in table.

Finally, Table 5 shows the level of agreement between the Actiheart and the SQUASH in categorizing people on the basis of fulfilling the NNGB. As with other outcome measures, there were no consistencies in findings on the basis of ethnicity. With the exception of Moroccan women, the SQUASH categorized a larger number of individuals as meeting the NNGB than did the Actiheart. Cohen’s kappa values were poor, the highest being 0.30 in Dutch women, while the lowest (0.08) was among African Surinamese men. We also looked at the percentage of agreement between the Actiheart and the SQUASH, correct classification was defined as both methods correctly classifying participants as either achieving or not achieving the NNGB. Using this approach 64.2 and 64.9% of the cases, among Dutch women and Moroccan men respectively were correctly classified. Among the other groups this was lower, varying from 45.2% in South Asian Surinamese men to 58.3% in Moroccan women.

**Table 5:** Categorization of participants on the basis of the Dutch norm for PA (NNGB).

		Dutch	South Asian Surinamese.	African Surinamese.	Turkish	Moroccan
**Women**	**Meets the norm SQUASH, n (%)**	41 (61)	22 (41)	20 (48)	14 (35)	7 (19)
	**Meets the norm Actiheart, n (%)**	29 (43)	16 (30)	14 (33)	10 (25)	14 (39)
	**Cohen’s kappa**	0.30	0.04	0.13	-0.06	0.04
	**% Agreement between Actiheart and SQUASH**^**a**^	64.2	55.6	57.1	55.0	58.3
**Men**	**Meets the norm SQUASH, n (%)**	29 (66)	25 (60)	23 (47)	26 (54)	17 (46)
	**Meets the norm Actiheart, n (%)**	23 (52)	10 (24)	15 (31)	13 (27)	12 (32)
	**Cohen’s kappa**	-0.02	0.00	0.08	0.08	0.28
	**% Agreement between Actiheart and SQUASH**^**a**^	50.0	45.2	55.1	52.1	64.9

^a^ Agreement means that participants are classified in the same way by the Actiheart and the SQUASH–for either meeting the norm or not meeting the norm

## Discussion

We aimed to validate self-reported PA using the SQUASH questionnaire in a multi-ethnic population and found that the SQUASH had a lower than expected test-re-test reliability in all ethnic groups. Compared to objective measures, the SQUASH largely underestimated the time spent in light intensity PA. Self-reported moderate and high intensity PA was higher than measured (overestimated), although differences at the group level were smaller than they were for light intensity PA. Nonetheless, wide limits of agreement (larger than 30 minutes per day) indicated poor validity at the individual level. The questionnaire also overestimated the number of participants that met the norm for PA. Validity coefficients were not lower in ethnic minority groups than in Dutch participants and we found no differences between the ethnic minority groups and Dutch population in the discrepancy between objectively and subjectively measured moderate/high intensity PA.

This is the first study to validate the SQUASH in a multi-ethnic population using an objective measure of PA, thus we are unable to compare our results with those among similar populations. A study by Wendel-Vos et al reported the SQUASH to be fairly reliable in a Dutch population, the Spearman’s correlation for test-re-test in that study was 0.58 (total PA). While we cannot directly compare our results due to methodological differences, our results indicate a somewhat lower reliability. Wendel-Vos et al included office workers (who are presumably higher educated) while we included a population-based sample of, on average, lower educated participants [10]. Socio-cultural background, sex, age, literacy and cognitive ability have been reported to influence reliability estimates of PA questionnaires [5]. Thus the lower test-re-test statistics in our study might reflect the ethnic and educational diversity of our population. The ICCs derived in our population for test-re-test reliability were also lower than what was reported in a recent review by Helmerhorst et al where median ICCs were 0.765 [5]. That review found reliability to be higher in studies with shorter test-re-test interval. We had a relatively long test-re-test interval of average 6–7 weeks. In designing the study we decided to use a longer time period for the test-re-test in order to minimize the chance that respondents remembered the answers they gave at the first administration as this may lead to an inflation of reliability estimates [24]. This might have increased the change that the second measurement in our study reflects real changes in the activity patterns over time in addition to measurement error and might be another reason for the low reliability statistics. Wendel-Vos et al also studied the validity of the SQUASH based on accelerometer data [10]. The Spearman’s correlation was 0.45 which is moderate and comparable to what we found among Dutch women (0.41) but higher than our findings among Dutch men (0.17). In our study, the Actiheart and the SQUASH consistently classified 50–64% of Dutch men and women respectively on the basis of the NNGB, which is lower the 69.5% reported by de Hollander et al in a similar study [11].

Consistent with our findings, two previous studies of the validity of PA questionnaires in multi-ethnic populations showed mixed results. In a Singapore study, Nang et al examined the validity of two different questionnaires (the long IPAQ and the SP2PAQ, a questionnaire based on several existing instruments) in three ethnic groups (Chinese, Malay and Indian) and found this to vary depending on the questionnaire studied as well as the outcome measure (either moderate or vigorous activity) [25]. In a Swedish study, Arvidsson et al found estimates to vary between Swedish and Iraqi participants, based on the outcome measure studied [26].

Our finding that compared to Actiheart measures, light intensity PA is underestimated with the SQUASH while moderate and high intensity activities are overestimated by all ethnic groups is consistent with studies among ‘general’ populations in different settings [6, 27–29]. Structured and more vigorous PA can be more easily recalled compared to more vaguely defined light PA like "Standing work, such as cooking, washing the dishes, ironing, feeding or bathing a child”. Additionally, the SQUASH was not designed to assess activities with an intensity of MET<2, which may explain the discrepancy with the Actiheart measures of light intensity activity in this study; the Actiheart provides specification for MET levels ≥1.5. Walking and cycling are measured in three domains by the SQUASH: during commuting; as a leisure-time activity; and, in the case of cycling, as a sport. Participants may inadvertently record these activities at multiple moments while filling in the questionnaire. The questionnaire was designed to be short, thus frequency, duration and intensity of each activity are asked in a single question. This means that participants have to consider the different dimensions of their routine PA simultaneously. Restructuring the questionnaire to take a more step-wise approach might improve reporting of PA, as might explicit inclusion of activities that are more commonly practiced by specific ethnic groups [30]. For example, dancing is popular among African origin Surinamese but may not be considered as a form of exercise by participants when filling in the questionnaire.

Our analysis indicated that the differences between reported and measured PA in the ethnic minority groups compared to Dutch participants were limited in the case of light intensity PA and not statistically significant in the case of moderate/high intensity PA, with the exception of Moroccan women. However, we observed large discrepancies in the categorization of participants in terms of meeting the NNGB, low Cohen’s kappa values indicate that the agreement between the two measures of PA was poor, in the Dutch as well as the ethnic minority groups. Our findings imply that the SQUASH does not correctly categorize individuals in terms of their PA level and thus does not provide a valid basis for the comparison of PA within or between different ethnic groups.

### Strengths and limitations

In the present study we used a combined accelerometer and heart rate sensor to validate the SQUASH. The Actiheart circumvents the limitations of accelerometers by providing indications of both time spent in physical activity and intensity levels [16]. It has been found to provide accurate measures of walking, running, sedentary, light- and moderate-intensity PAs in adults [16, 31, 32]. Individual calibration improves energy expenditure estimates and as the Actiheart is worn continuously during the measurement period, including during showering, water sports and sleeping, it provides a more complete picture of PA. However, despite the fact that the Actiheart can measure activity intensity, it does not provide information about the location or purpose of individual activities.

There are also some potential limitations, firstly, due to our study design. PA measures using the Actiheart may not be representative of habitual PA as the devices were worn for a maximum of 5 days during a single period. This decision was based on practical considerations as well as a desire to limit respondent burden. Despite the fact that weekend days were included in the study period, a full week might have better captured habitual PA. Additionally, the SQUASH was completed prior to wearing the Actiheart; differences between the measures may be due to true differences in PA during the week that participants wore the Actiheart, an alternative study design in which participants completed the SQUASH for the week in which they wore the Actiheart may have been preferable. Furthermore, within-individual seasonal differences in PA were not assessed [33]. Secondly, the response rate was low among some ethnic groups which may affect the external validity of our findings; this response rate is similar to what is commonly seen in studies among ethnic minority groups [34]. Education, language and literacy levels are likely to be important confounders, particularly as in this study the SQUASH was self-administered by participants. We did not assess level of literacy, but we found no difference in response on the basis of education level. However, we cannot rule out that language formed a barrier for response among Moroccan participants as we did not provide a translated version of the questionnaire. Thirdly, considerably fewer participants among the ethnic minorities filled in the SQUASH a second time, thus the sample size for the test-re-test reliability analyses was poor to fair. Higher reporting of PA at the second time point may be due to participants being made aware of their PA, as a result of taking part in this study. To minimize this potential we gave feedback on PA levels only after full completion of the study.

## Conclusions

We found considerable variation in the reliability and validity of the SQUASH with no consistent patterns on the basis of ethnicity. Although the ethnic minority participants were not any worse at reporting PA than were the Dutch origin men and women, our findings imply that the SQUASH does not provide a valid basis for the comparison of PA between different ethnic groups.

## Supporting Information

S1 File Figs A and B. Bland Altman Plots for Light Intensity Physical Activity.
(PDF)
