# Supplementary figures and images for: Validation of the SQUASH Physical Activity Questionnaire in a Multi-Ethnic Population: The HELIUS Study
Source: PLoS One. 2016 Aug 30;11(8):e0161066. doi: 10.1371/journal.pone.0161066 (PMC5004804; doi:10.1371/journal.pone.0161066)

S1: Bland Altman Plots for Light Intensity Physical Activity

Figure A: Men

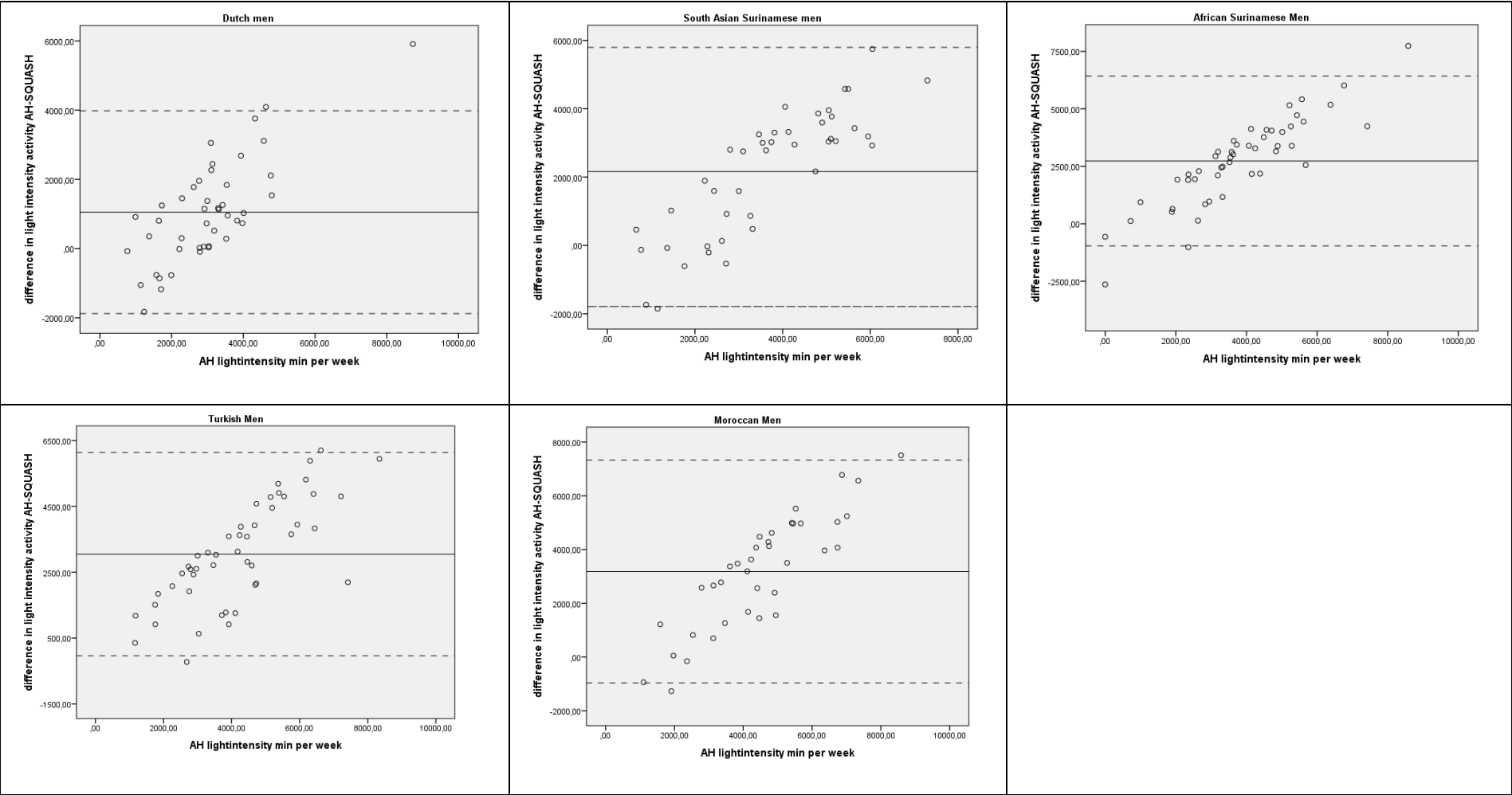

Figure B: Women

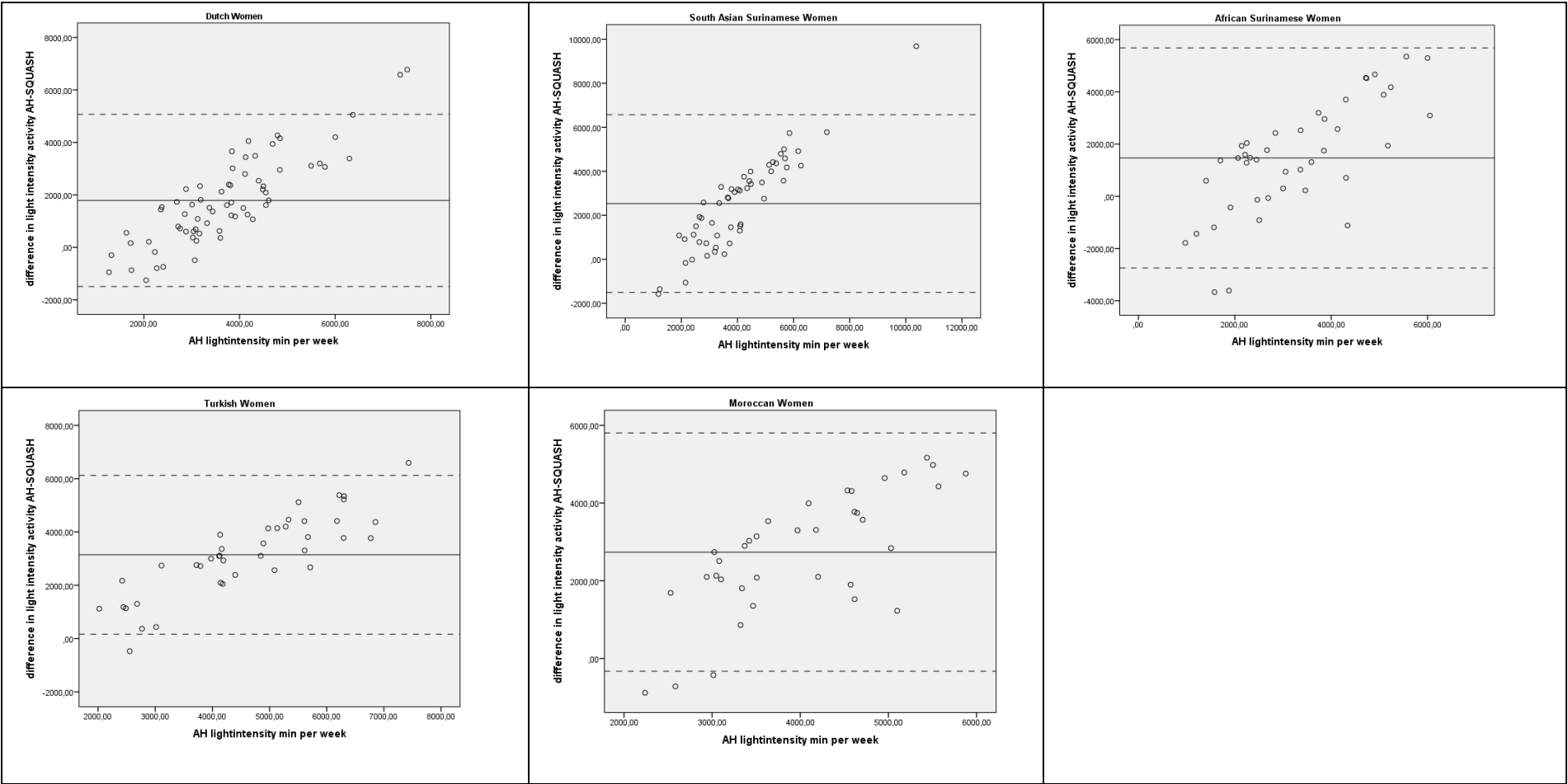

Supplement: S1 File — Figs A and B. Bland Altman Plots for Light Intensity Physical Activity. (PDF) [file pone.0161066.s001.pdf]
